# Calculating and reporting effect sizes to facilitate cumulative science: a practical primer for *t*-tests and ANOVAs

**DOI:** 10.3389/fpsyg.2013.00863

**Published:** 2013-11-26

**Authors:** Daniël Lakens

**Affiliations:** Human Technology Interaction Group, Eindhoven University of TechnologyEindhoven, Netherlands

**Keywords:** effect sizes, power analysis, cohen's *d*, eta-squared, sample size planning

## Abstract

Effect sizes are the most important outcome of empirical studies. Most articles on effect sizes highlight their importance to communicate the practical significance of results. For scientists themselves, effect sizes are most useful because they facilitate cumulative science. Effect sizes can be used to determine the sample size for follow-up studies, or examining effects across studies. This article aims to provide a practical primer on how to calculate and report effect sizes for *t*-tests and ANOVA's such that effect sizes can be used in a-priori power analyses and meta-analyses. Whereas many articles about effect sizes focus on between-subjects designs and address within-subjects designs only briefly, I provide a detailed overview of the similarities and differences between within- and between-subjects designs. I suggest that some research questions in experimental psychology examine inherently intra-individual effects, which makes effect sizes that incorporate the correlation between measures the best summary of the results. Finally, a supplementary spreadsheet is provided to make it as easy as possible for researchers to incorporate effect size calculations into their workflow.

Effect sizes are the most important outcome of empirical studies. Researchers want to know whether an intervention or experimental manipulation has an effect greater than zero, or (when it is obvious an effect exists) how big the effect is. Researchers are often reminded to report effect sizes, because they are useful for three reasons. First, they allow researchers to present the magnitude of the reported effects in a standardized metric which can be understood regardless of the scale that was used to measure the dependent variable. Such standardized effect sizes allow researchers to communicate the practical significance of their results (what are the practical consequences of the findings for daily life), instead of only reporting the statistical significance (how likely is the pattern of results observed in an experiment, given the assumption that there is no effect in the population). Second, effect sizes allow researchers to draw meta-analytic conclusions by comparing standardized effect sizes across studies. Third, effect sizes from previous studies can be used when planning a new study. An a-priori power analysis can provide an indication of the average sample size a study needs to observe a statistically significant result with a desired likelihood.

The aim of this article is to explain how to calculate and report effect sizes for differences between means in between and within-subjects designs in a way that the reported results facilitate cumulative science. There are some reasons to assume that many researchers can improve their understanding of effect sizes. For example, researchers predominantly report the effect size partial eta squared (η^2^_*p*_), which is provided by statistical software packages such as SPSS. The fact that η^2^_*p*_ is often reported for One-Way ANOVAs (where partial eta squared equals eta squared), indicates that researchers are either very passionate about unnecessary subscript letters, or rely too much on the effect sizes as they are provided by statistical software packages.

This practical primer should be seen as a complementary resource for psychologists who want to learn more about effect sizes (for excellent books that discuss this topic in more detail, see Cohen, 1988; Maxwell and Delaney, 2004; Grissom and Kim, 2005; Thompson, 2006; Aberson, 2010; Ellis, 2010; Cumming, 2012; Murphy et al., 2012). A supplementary spreadsheet is provided to facilitate effect size calculations. Reporting standardized effect sizes for mean differences requires that researchers make a choice about the standardizer of the mean difference, or a choice about how to calculate the proportion of variance explained by an effect. In this article, these choices will be highlighted for Cohen's *d* and eta squared (η^2^), two of the most widely used effect sizes in psychological research, with a special focus on the difference between within and between-subjects designs. I point out some caveats for researchers who want to perform power-analyses for within-subjects designs, and provide recommendations regarding the effect sizes that should be reported.

Knowledge about the expected size of an effect is important information when planning a study. Researchers typically rely on null hypothesis significance tests to draw conclusions about observed differences between groups of observations. The probability of correctly rejecting the null hypothesis is known as the *power* of a statistical test (Cohen, 1988). Statistical power depends on the sample size of the study (through its influence on the reliability of the sample values, and specifically the extent to which sample values can be expected to be an approximation of the population values), the size of the effect, and the significance criterion (typically α = 0.05). If three are known (or estimated), the fourth parameter can be calculated. In an a-priori power analysis, researchers calculate the sample size needed to observe an effect of a specific size, with a pre-determined significance criterion, and a desired statistical power.

A generally accepted minimum level of power is 0.80 (Cohen, 1988). This minimum is based on the idea that with a significance criterion of 0.05 the ratio of a Type 2 error (1-power) to a Type 1 error is 0.20/.05, so concluding there *is* an effect when there is *no* effect in the population is considered four times as serious as concluding there is *no* effect when there *is* an effect in the population. Some researchers have argued that Type 2 errors can potentially have much more serious consequences than Type 1 errors, however (Fiedler et al., 2012). Thus, although a power of 0.80 is the recommended minimum, higher power (e.g., 0.95) is more desirable, as long as it is practically feasible. Effect size estimates have their own confidence intervals [for calculations for Cohen's *d*, see Cumming (2012), for *F*-tests, see Smithson (2001)], which are often very large in experimental psychology. Therefore, researchers should realize that the confidence interval around a sample size estimate derived from a power analysis is often also very large, and might not provide a very accurate basis to determine the sample size of a future study. Meta-analyses can provide more accurate effect size estimates for power analyses, and correctly reporting effect size estimates can facilitate future meta-analyses [although due to publication bias, meta-analyses might still overestimate the true effect size, see Brand et al. (2008); Bakker et al. (2012)].

## Statistical significance and generalizability of effect size estimates

Consider two sets of observations with *M*_1_ = 7.7, *SD*_1_ = 0.95, and *M*_2_ = 8.7, *SD*_2_ = 0.82. Depending on whether the data were collected in a between or within-subjects design, the effect size partial eta squared (η^2^_*p*_) for the difference between these two observations (for details, see the illustrative example below) is either 0.26 or 0.71, respectively. Given that the mean difference is the same (i.e., 1) regardless of the design, which of these two effect sizes is the “true” effect size? There are two diverging answers to this question. One viewpoint focusses on the generalizability of the effect size estimate across designs, while the other viewpoint focusses on the statistical significance of the difference between the means. I will briefly discuss these two viewpoints.

As Maxwell and Delaney (2004, p. 548) remark: “a major goal of developing effect size measures is to provide a standard metric that meta-analysts and others can interpret across studies that vary in their dependent variables as well as types of designs.” This first viewpoint, which I will refer to as the *generalizable effect size estimate* viewpoint, assumes that it does not matter whether you use a within-subjects design or a between-subjects design. Although you can exclude individual variation in the statistical test if you use a pre- and post-measure, and the statistical power of a test will often substantially increase, the effect size (e.g., η^2^_*p*_) should not differ depending on the design that was used. Therefore, many researchers regard effect sizes in within-subjects designs as an overestimation of the “true” effect size (e.g., Dunlap et al., 1996; Olejnik and Algina, 2003; Maxwell and Delaney, 2004).

A second perspective, which I will refer to as the *statistical significance* viewpoint, focusses on the statistical test of a predicted effect, and regards individual differences as irrelevant for the hypothesis that is examined. The goal is to provide statistical support for the hypothesis, and being able to differentiate between variance that is due to individual differences and variance that is due to the manipulation increases the power of the study. Researchers advocating the statistical significance viewpoint regard the different effect sizes (e.g., η^2^_*p*_) in a within- compared to between-subjects design as a benefit of a more powerful design. The focus on the outcome of the statistical test in this perspective can be illustrated by the use of confidence intervals. As first discussed by Loftus and Masson (1994), the use of traditional formulas for confidence intervals (developed for between-subjects designs) can result in a marked discrepancy between the statistical summary of the results and the error bars used to visualize the differences between observations. To resolve this inconsistency, Loftus and Masson (1994, p. 481) suggest that: “Given the irrelevance of intersubject variance in a within-subjects design, it can legitimately be ignored for purposes of statistical analysis.”

To summarize, researchers either focus on generalizable effect size estimates, and try to develop effect size measures that are independent from the research design, or researchers focus on the statistical significance, and prefer effect sizes (and confidence intervals) to reflect the conclusions drawn by the statistical test. Although these two viewpoints are not mutually exclusive, they do determine some of the practical choices researchers make when reporting their results. Regardless of whether researchers focus on statistical significance or generalizability of measurements, cumulative science will benefit if researchers determine their sample size a-priori, and report effect sizes when they share their results. In the following sections, I will discuss how effect sizes to describe the differences between means are calculated, with a special focus on the similarities and differences in within and between-subjects designs, followed by an illustrative example.

## Differences and similarities between effect sizes

As Poincaré (1952, p. 34) has said: “mathematics is the art of giving the same name to different things.” Unfortunately, in the domain of effect size calculations statisticians have failed Poincare. Effect sizes have either different names although they are basically the same entity (such as referring to *r*^2^ as η^2^), or they have received the same name, despite being calculated in different ways (such as referring to an effect size as Cohen's *d*, regardless of the way it is calculated). Effect sizes can be grouped in two families (Rosenthal, 1994): The *d* family (consisting of standardized mean differences) and the *r* family (measures of strength of association). Conceptually, the *d* family effect sizes are based on the difference between observations, divided by the standard deviation of these observations. The *r* family effect sizes describe the proportion of variance that is explained by group membership [e.g., a correlation (*r*) of 0.5 indicates 25% (*r*^2^) of the variance is explained by the difference between groups]. These effect sizes are calculated from the sum of squares (the difference between individual observations and the mean for the group, squared, and summed) for the effect divided by the sums of squares for other factors in the design.

A further differentiation between effect sizes is whether they correct for bias or not (e.g., Thompson, 2007). Population effect sizes are almost always estimated on the basis of samples, and all population effect size estimates based on sample averages overestimate the true population effect (for a more detailed explanation, see Thompson, 2006). Therefore, corrections for bias are used (even though these corrections do not always lead to a completely unbiased effect size estimate). In the *d* family of effect sizes, the correction for Cohen's *d* is known as Hedges' *g*, and in the *r* family of effect sizes, the correction for eta squared (η^2^) is known as omega squared (ω^2^). These effects sizes will be discussed in more detail in the following paragraphs.

### Cohen's d in between-subjects designs

Cohen's *d* is used to describe the standardized mean difference of an effect. This value can be used to compare effects across studies, even when the dependent variables are measured in different ways, for example when one study uses 7-point scales to measure dependent variables, while the other study uses 9-point scales, or even when completely different measures are used, such as when one study uses self-report measures, and another study used physiological measurements. It ranges from 0 to infinity. Cohen (1988) uses subscripts to distinguish between different versions of Cohen's *d*, a practice I will follow because it prevents confusion (without any subscript, Cohen's *d* denotes the entire family of effect sizes). Cohen refers to the standardized mean difference between two groups of independent observations for the *sample* as *d_s_* which is given by:

(1) $$d_{s}=\frac{{\bar{X}}_{1}-{\bar{X}}_{2}}{\sqrt{\frac{\left(n_{1}-1\right)SD_{1}^{2}+\left(n_{2}-1\right)SD_{2}^{2}}{n_{1}+n_{2}-2}}}$$

In this formula, the numerator is the difference between means of the two groups of observations. The denominator is the pooled standard deviation. Remember that the standard deviation is calculated from the differences between each individual observation and the mean for the group. These differences are squared to prevent the positive and negative values from cancelling each other out, and summed (also referred to as the *sum of squares*). This value is divided by the number of observations minus one, which is Bessel's correction for bias in the estimation of the population variance, and finally the square root is taken. This correction for bias in the sample estimate of the population variance is based on the least squares estimator (see McGrath and Meyer, 2006). Note that Cohen's *d_s_* is sometimes referred to as Cohen's *g*, which can be confusing. Cohen's *d_s_* for between-subjects designs is directly related to a *t*-test, and can be calculated by:

(2) $$d_{s}=t\sqrt{\frac{1}{n_{1}}+\frac{1}{n_{2}}}$$

If only the total sample size is known, Cohen's $d_{s}\approx 2\times t/\sqrt{N}$. Statistical significance is typically expressed in terms of the height of *t*-values for specific sample sizes (but could also be expressed in terms of whether the 95% confidence interval around Cohen's *d_s_* includes 0 or not), whereas Cohen's *d_s_* is typically used in an a-priori power analysis for between-subjects designs (even though a power analysis could also be based on the *t*-value and *n* per condition). Formula 2 underlines the direct relation between the effect size and the statistical significance.

The standardized mean difference can also be calculated without Bessel's correction, in which case it provides the maximum likelihood estimate for a sample, as noted by Hedges and Olkin (1985). The difference between Cohen's *d_s_* and Cohen's *d*_pop_ (for the population) is important to keep in mind when converting Cohen's *d*_*s*_ to the point biserial correlation *r*_*pb*_ (which will simply be referred to as *r* in the remainder of this text). Many textbooks provide the formula to convert Cohen's *d*_pop_ to *r*, while the formula to convert Cohen's *d_s_* to *r* (which can only be used for between-subjects designs) is provided by McGrath and Meyer (2006):

(3) $$r=\frac{d_{s}}{\sqrt{d_{s}^{2}{}^{+}\frac{N^{2}-2N}{n_{1}n_{2}}}}$$

As mentioned earlier, the formula for Cohen's *d_s_*, which is based on sample averages gives a biased estimate of the population effect size (Hedges and Olkin, 1985), especially for small samples (*n* < 20). Therefore, Cohen's *d_s_* is sometimes referred to as the *uncorrected effect size*. The *corrected effect size*, or Hedges's *g* (which is unbiased, see Cumming, 2012), is:

(4) $$\text{Hedges}\prime \text{s }g_{s}=\text{Cohen}\prime \text{s }d_{s}\times \left(1-\frac{3}{4\left(n_{1}+n_{2}\right)-9}\right)$$

I use the same subscript letter in Hedges's *g* to distinguish different calculations of Cohen's *d*. Although the difference between Hedges's *g_s_* and Cohen's *d_s_* is very small, especially in sample sizes above 20 (Kline, 2004), it is preferable (and just as easy) to report Hedges's *g_s_*. There are also bootstrapping procedures to calculate Cohen's *d_s_* when the data are not normally distributed, which can provide a less biased point estimate (Kelley, 2005). As long as researchers report the number of participants in each condition for a between-subjects comparison and the *t*-value, Cohen's *d* and Hedges' *g* can be calculated. Whenever standard deviations differ substantially between conditions, Glass's Δ should be reported (see below).

### Interpreting cohen's *d*

How should researchers interpret this effect size? A commonly used interpretation is to refer to effect sizes as small (*d* = 0.2), medium (*d* = 0.5), and large (*d* = 0.8) based on benchmarks suggested by Cohen (1988). However, these values are arbitrary and should not be interpreted rigidly (Thompson, 2007). Small effect sizes can have large consequences, such as an intervention that leads to a reliable reduction in suicide rates with an effect size of *d* = 0.1. The only reason to use these benchmarks is because findings are extremely novel, and cannot be compared to related findings in the literature (Cohen, 1988). Cohen's *d* in between-subject designs can be readily interpreted as a percentage of the standard deviation, such that a Cohen's *d* of 0.5 means the difference equals half a standard deviation. However, the best way to interpret Cohen's *d* is to relate it to other effects in the literature, and if possible, explain the practical consequences of the effect. Regrettably, there are no clear recommendation of how to do so (Fidler, 2002).

An interesting, though not often used, interpretation of differences between groups can be provided by the *common language effect size* (McGraw and Wong, 1992), also known as the probability of superiority (Grissom and Kim, 2005), which is a more intuitively understandable statistic than Cohen's *d* or *r*. It can be calculated directly from Cohen's *d*, converts the effect size into a percentage, and expresses the probability that a randomly sampled person from one group will have a higher observed measurement than a randomly sampled person from the other group (for between designs) or (for within-designs) the probability that an individual has a higher value on one measurement than the other. It is based on the distribution of the difference scores, with a mean that is estimated from the mean differences between the samples, and a standard deviation that is the square root of the sum of the sample variances divided by two. Mathematically, the common language effect size is the probability of a Z-score greater than the value that corresponds to a difference between groups of 0 in a normal distribution curve. Z can be calculated by:

(5) $$Z=\frac{\left|X_{1}-X_{2}\right|}{\sqrt{\frac{SD_{1}^{2}+SD_{2}^{2}}{2}}}$$

after which the common language effect size is the percentage associated with the upper tail probability of this value. The supplementary spreadsheet provides an easy way to calculate the common language effect size.

### Cohen's *d* in one-sample or correlated samples comparisons

Conceptually, calculating Cohen's *d* for correlated measurements is the same as calculating Cohen's *d* for independent groups, where the differences between two measurements are divided by the standard deviation of both groups of measurements. However, in the case of correlated measurements the dependent *t*-test uses the standard deviation of the difference scores. Testing whether observations from two correlated measurements are significantly different from each other using a paired samples *t*-test is mathematically identical to testing whether the difference scores of the correlated measurements is significantly different from 0 using a one-sample *t*-test. Similarly, calculating the effect size for the difference between two correlated measurements is similar to the effect size that is calculated for a one sample *t*-test. The standardized mean difference effect size for within-subjects designs is referred to as Cohen's *d_z_*, where the Z alludes to the fact that the unit of analysis is no longer X or Y, but their difference, Z, and can be calculated with:

(6) $$\text{Cohen}\prime \text{s }d_{z}=\frac{M_{\text{diff}}}{\sqrt{\frac{\sum {\left(X_{\text{diff}}-M_{\text{diff}}\right)}^{2}}{N-1}}}$$

where the numerator is the difference between the mean (*M*) of the difference scores and the comparison value μ (e.g., 0), and the denominator is the standard deviation of the difference scores (*S_diff_*). The effect size estimate Cohen's *d_z_* can also be calculated directly from the *t*-value and the number of participants using the formula provided by Rosenthal (1991):

(7) $$\text{Cohen}\prime \text{s }d_{z}=\frac{t}{\sqrt{n}}$$

Given the direct relationship between the *t*-value of a paired-samples *t*-test and Cohen's *d_z_*, it will not be surprising that software that performs power analyses for within-subjects designs (e.g., G^*^Power, (Faul et al., 2009)) relies on Cohen's *d_z_* as input. To allow researchers to perform an a-priori power analysis, it is therefore enough to report the *t*-value and the number of pairs of observations (or the degrees of freedom, *n* − 1). Cohen's *d_z_* is only rarely used in meta-analyses, because researchers often want to be able to compare effects across within and between-subject designs. One solution (which is not generally recommended) is to use Cohen's *d_rm_*, where the subscript is used by Morris and DeShon (2002) to indicate this is the equivalent of Cohen's *d* for *repeated measures*. Cohen's *d_rm_* controls for the correlation between the two sets of measurements, as explained below.

An alternative formula to calculate the standard deviation of the difference scores from the standard deviations of both groups and their correlation is given by Cohen (1988) as:

(8) $$S_{diff}=\sqrt{SD_{1}^{2}+SD_{2}^{2}-2\times r\times SD_{1}\times SD_{2}}$$

where *r* is the correlation between measures, and *S* is the standard deviation within each of the two sets of observations. As the correlation between measures increases, the standard deviation of the difference scores decreases. In experimental psychology, correlations between measures are typically a positive non-zero value. This has two consequences. First, within-subjects designs typically have more statistical power than between-subjects designs, because the standard deviation of the difference scores is smaller than the standard deviations of the two groups of observations. Second, under the assumption of equal variances (for unequal variances, Glass's Δ should be calculated, see below), the mean standardized difference between the two correlated measurements is standardized by a value which is $\sqrt{2(1-r)}$ larger than the standard deviation for independent observations (see Cohen, 1988), and thus:

(9) $$\text{Cohen}\prime \text{s }d_{rm}=\frac{M_{\text{diff}}}{\sqrt{SD_{1}^{2}+SD_{2}^{2}-2\times r\times SD_{1}\times SD_{2}}}\times \sqrt{2(1-r)}$$

When *r* = 0.5 and the standard deviations in both groups of measurements are the same, Cohen's *d_s_* from a between-subjects design and Cohen's *d_rm_* from a within-subjects design are identical, but differences in the standard deviations between the two groups will introduce differences between the two effect sizes, which become more pronounced when *r* approaches 0 or 1.

Another solution to calculate Cohen's *d* for within-subjects designs is to simply use the *average* standard deviation of both repeated measures as a standardizer (which ignores the correlation between the measures). Cumming (2012) refers to this approach as Cohen's *d_av_*, which is simply:

(10) $$\text{Cohen}\prime \text{s }d_{av}=\frac{M_{\text{diff}}}{\frac{SD_{1}+SD_{2}}{2}}$$

When the standard deviations of both groups of observations are equal, Cohen's *d_av_*, and Cohen's *d_rm_* are identical, and the effect size equals Cohen's *d_s_* for the same means and standard deviations in a between subject design. In general, Cohen's *d_av_* will be more similar to Cohen's *d_s_* (compared to Cohen's *d_rm_*), except when correlations between measures are low, and the difference between the standard deviations is large. Cohen's *d_rm_* is always more conservative, but with high correlations between observations, sometimes unreasonably conservative.

When *r* is larger than 0.5, Cohen's *d_z_* will be larger than Cohen's *d_rm_* and Cohen's *d_av_*, and when *r* is smaller than 0.5, Cohen's *d_z_* will be smaller than Cohen's *d_rm_* and Cohen's *d_av_* (Morris and DeShon, 2002). Dunlap et al. (1996) argue against reporting Cohen's *d_z_* based on the idea that the correlation between measures does not change the size of the effect, but merely makes it more noticeable by reducing the standard error, and therefore refer to Cohen's *d_z_* as an *overestimation* of the effect size. Although Cohen's *d_z_* is rarely reported as an effect size, there are some situations when I believe it to be perfectly defensible (see the General Discussion). However, I would in general recommend to report effect sizes that cannot be calculated from other information in the article, and that are widely used so that most readers should understand them. Because Cohen's *d_z_* can be calculated from the *t*-value and the *n*, and is not commonly used, my general recommendation is to report Cohen's *d_rm_* or Cohen's *d_av_*.

Because Cohen's *d_rm_* and Cohen's *d_av_* are based on sample estimates, and these are positively biased, we should apply Hedges' correction. However, unlike Hedges's *g_s_*, Hedges's *g_av_* Hedges's *g_rm_* are not completely unbiased (Cumming, 2012). After entering the required information in the supplementary spreadsheet, it recommends either Hedges's *g_av_* or Hedges's *g_rm_* based on which of these two values is most similar to Cohen's *d_s_* in a between subjects design (in line with the goal to report an effect size estimate that is comparable across within and between participant designs).

In some designs there are good reasons to believe the manipulation did not only influence the mean between observations, but also influenced the standard deviation. For example, pre- and post-measurements in a study that examines an intervention might differ in their standard deviation as a consequence of the intervention. In such designs, Glass et al. (1981) recommends to use either the standard deviation of the pre-measurement as a standardizer (often recommended, and used in the supplementary spreadsheet) or the standard deviation of the post-measurement. This is referred to as Glass's Δ (and subscripts can be used to indicate whether the pre- or post-measurement standard deviation was used). These options highlight the importance of specifying which version of the effect size *d* is calculated, and the use of subscript letters might be an efficient way to communicate the choices made. Researchers have to choose which effect size is the best representation of the effect they are interested in. Table 1 summarizes when different versions of effect size measures in the *d* family are used. The common language effect size can be reported in addition to Cohen's *d* to facilitate the interpretation of the effect size.

**Table 1:** **Summary of d family effect sizes, standardizers, and their recommended use**.

**ES**	**Standardizer**	**Use**
Cohen's *d*_pop_	σ (population)	Independent groups, use in power analyses when population σ is known, σ calculated with *n*
Cohen's *d_s_*	Pooled *SD*	Independent groups, use in power analyses when population σ is unknown, σ calculated with *n*-1
Hedges' *g*	Pooled *SD*	Independent groups, corrects for bias in small samples, report for use in meta-analyses
Glass's Δ	*SD* pre measurement or control condition	Independent groups, use when experimental manipulation might affect the *SD*
Hedges' *g_av_*	(*SD*_1_ + *SD*_2_)/2	Correlated groups, report for use in meta-analyses (generally recommended over Hedges' *g_rm_*)
Hedges' *g_rm_*	*SD* difference scores corrected for correlation	Correlated groups, report for use in meta-analyses (more conservative then Hedges' *g_av_*)
Cohen's *d_z_*	*SD* difference scores	Correlated groups, use in power analyses

### Eta-squared in between and within-subjects comparisons

Eta squared η^2^ (part of the *r* family of effect sizes, and an extension of *r*^2^ that can be used for more than two sets of observations) measures the proportion of the variation in Y that is associated with membership of the different groups defined by X, or the sum of squares of the effect divided by the total sum of squares:

(11) $$\eta^{2}=\frac{SS_{\text{effect}}}{SS_{\text{total}}}$$

An η^2^ of 0.13 means that 13% of the total variance can be accounted for by group membership. Although η^2^ is an efficient way to compare the sizes of effects *within* a study (given that every effect is interpreted in relation to the total variance, all η^2^ from a single study sum to 100%), eta squared cannot easily be compared *between* studies, because the total variability in a study (*SS*_total_) depends on the design of a study, and increases when additional variables are manipulated. Keppel (1991) has recommended partial eta squared (η^2^_*p*_) to improve the comparability of effect sizes between studies, which expresses the sum of squares of the effect in relation to the sum of squares of the effect and the sum of squares of the error associated with the effect. Partial eta squared is calculated as:

(12) $$\eta_{p}^{2}=\frac{SS_{\text{effect}}}{SS_{\text{effect}}+SS_{\text{error}}}$$

For designs with fixed factors (manipulated factors, or factors that exhaust all levels of the independent variable, such as alive vs. dead), but *not* for designs with measured factors or covariates, partial eta squared can be computed from the *F*-value and its degrees of freedom (e.g., Cohen, 1965):

(13) $$\eta_{p}^{2}=\frac{F\times df_{\text{effect}}}{F\times df_{\text{effect}}+df_{\text{error}}}$$

For example, for an *F*_(1, 38)_ = 7.21, η^2^_*p*_ = 7.21 × 1/(7.21 × 1 + 38) = 0.16. This relationship between η^2^_*p*_ and *F* illustrates how η^2^_*p*_ can be used in power analyses to estimate the desired sample size for a future experiment, and software programs such as G^*^Power require η^2^_*p*_ as input for this reason. If researchers want to facilitate power analyses, they should report η^2^_*p*_, especially for designs where not all factors are manipulated.

Users of G^*^Power should be aware that the default η^2^_*p*_ for within designs as used by G^*^Power does not correspond to the η^2^_*p*_ as provided by SPSS. When using η^2^_*p*_ as provided by SPSS to perform power calculations in G^*^Power, one cannot simply use the default settings of the program. Where SPSS provides a η^2^_*p*_ that already incorporates the correlation between paired measures (hence the difference in η^2^_*p*_ for the same two means and standard deviations depending on whether they come from a between or within-subjects designs), G^*^Power defines η^2^_*p*_ for within-subjects designs in exactly the same way as for between-subjects designs (and incorporates the correlations between dependent measures during the power calculations). A more formal description of these differences, as well as an explanation how to convert SPSS η^2^_*p*_ to G^*^Power η^2^_*p*_ is provided in the Appendix. The most recent version of G^*^Power (3.1) allows researchers to indicate that they are directly using an SPSS η^2^_*p*_ in their calculations by selecting a radio button in an options menu. This option is not the default, and it is likely that researchers will calculate a wrong sample estimate if they are not aware of the difference between SPSS η^2^_*p*_ and G^*^Power η^2^_*p*_. When η^2^_*p*_ is used in the remainder of this document, the SPSS equivalent that includes the correlation between dependent measures is meant.

Although η^2^_*p*_ is more useful when the goal is to compare effect sizes across studies, it is not perfect, because η^2^_*p*_ differs when the same two means are compared in a within-subjects design or a between-subjects design. In a within-subjects ANOVA, the error sum of squares can be calculated around the mean of each measurement, but also around the mean of each individual when the measurements are averaged across individuals. This allows researchers to distinguish variability due to individual differences from variability due to the effect in a within-subjects design, whereas this differentiation is not possible in a between-subjects design. As a consequence, whenever the two groups of observations are positively correlated, η^2^_*p*_ will be larger in a within-subjects design than in a between-subjects design. This is also the reason a within-subjects ANOVA typically has a higher statistical power than a between-subjects ANOVA.

Olejnik and Algina (2003) provide further reasons why η^2^_*p*_ can only be used to compare effects between studies with similar experimental designs. Differences in the inclusion of covariates or blocking factors between experimental designs (for example, including the gender of participants in the analysis as a between-subjects factor, which will account for some of the variance) can influence the size of η^2^_*p*_. They propose generalized eta squared (η^2^_*G*_), which excludes variation from other factors from the effect size calculation (to make the effect size comparable with designs in which these factors were not manipulated), but includes variance due to individual differences (to make the effect size comparable with between-subjects designs where this individual variance cannot be controlled for). When all factors are manipulated between participants η^2^_*G*_ and η^2^_*p*_ are identical. In other experimental designs, η^2^_*G*_ can be computed from the output of an ANOVA, and the supplementary spreadsheet allows researchers to easily calculate η^2^_*G*_ for the most commonly used experimental designs.

As mentioned before, eta squared is an uncorrected effect size estimate that estimates the amount of variance explained based on the sample, and not based on the entire population. Omega squared (ω^2^) has been suggested to correct for this bias (Hayes, 1963), even though it is at best a less biased estimate (Winkler and Hays, 1975). As with Hedges' correction for Cohen's *d*, providing ω^2^ instead of η^2^ is formally correct. However, the difference is typically small, and the bias decreases as the sample size increases. In between-subjects designs with fixed factors ω^2^ and ω^2^_*p*_ can be calculated through the formulas provided by Olejnik and Algina (2000) and Bakeman (2005):

(14) $$\omega^{2}=\frac{df_{\text{effect}}\times (MS_{\text{effect}}-MS_{\text{error}})}{SS_{\text{total}}+MS_{\text{error}}}$$

(15) $$\omega_{p}^{2}=\frac{df_{\text{effect}}\times (MS_{\text{effect}}-MS_{\text{error}})}{df_{\text{effect}}\times MS_{\text{effect}}+(N-df_{\text{effect}})\times MS_{\text{error}}}$$

For within-subjects designs, ω^2^_*p*_ is calculated in the same way as for between-subjects designs (see above), but ω^2^ is calculated by:

(16) $$\omega^{2}=\frac{df_{\text{effect}}\times (MS_{\text{effect}}-MS_{\text{error}})}{SS_{\text{total}}+MS_{\text{subjects}}}$$

Calculating generalized omega squared (ω^2^_*G*_) can become rather complex, depending on the design (see the lists of formulas provided by Olejnik and Algina, 2003). Given this complexity, and the relatively small difference between the bias and less biased estimate, I recommend researchers report η^2^_*G*_ and/or η^2^_*p*_, at least until generalized omega-squared is automatically provided by statistical software packages. For designs where all factors are manipulated between participants, η^2^_*p*_ and η^2^_*G*_ are identical, so either effect size can be reported. For within-subjects designs and mixed designs where all factors are manipulated, η^2^_*p*_ can always be calculated from the *F*-value and the degrees of freedom using formula 13, but η^2^_*G*_ cannot be calculated from the reported results, and therefore I recommend reporting η^2^_*G*_ for these designs (but providing η^2^_*p*_ in addition to η^2^_*G*_ would be a courtesy to readers). The supplementary spreadsheet provides a relatively easy way to calculate η^2^_*G*_ for commonly used designs. For designs with measured factors or covariates, neither η^2^_*p*_ nor η^2^_*G*_ can be calculated from the reported results, and thus I recommend reporting both η^2^_*p*_ as η^2^_*G*_, where the first can be used in power analyses, and the second can be used in meta-analyses or interpreted against the benchmarks provided by Cohen (1988). Table 2 summarizes when different versions of effect size measures in the *r* family are used.

**Table 2:** **Summary of *r* family effect sizes and their recommended use**.

**ES (Biased)**	**ES (Less Biased)**	**Use**
eta squared (μ^2^)	omega squared (ω^2^)	Use for comparisons of effects within a single study
eta squared (μ^2^_*p*_)	omega squared (ω^2^_*p*_)	Use in power analyses, and for comparisons of effect sizes across studies with the same experimental design.
Generalized eta squared (μ^2^_*G*_)	Generalized omega squared (ω^2^_*G*_)	Use in meta-analyses to compare across experimental designs

Cohen (1988) has provided benchmarks to define small (η^2^ = 0.01), medium (η^2^ = 0.06), and large (η^2^ = 0.14) effects. As Olejnik and Algina (2003) explain, these benchmarks were developed for comparisons between unrestricted populations (e.g., men vs. women), and using these benchmarks when interpreting the η^2^_*p*_ effect size in designs that include covariates or repeated measures is not consistent with the considerations upon which the benchmarks were based. Although η^2^_*G*_ can be compared against the benchmarks provided by Cohen (1988), this should only be done as a last resort, and it is preferable to relate the effect size to other effects in the literature (Thompson, 2007). The common language effect size can be calculated for contrasts from the means and standard deviations of the two measurements as explained for the dependent and independent *t*-tests above. This concludes the general summary of how to calculate and report effect sizes. To highlight some more practical considerations, I will provide an example in which the same two sets of observations are analyzed using paired and independent *t*-tests, as well as One-way and repeated measures ANOVAs.

## An illustrative example

In this example, I will address some practical considerations by analyzing the dataset in Table 3, which contains two sets of observations. This data will be analyzed in two ways, either as a between design or as a within design. We will assume that Movie 1 and Movie 2 are movie evaluations for two different movies on a scale from 1 (very bad) to 10 (very good). First, let's consider a situation where these movie evaluations are collected from two different groups. An independent *t*-test would provide *t*_(18)_ = 2.52, *p* = 0.022 (note that the supplementary spreadsheet also provides the outcome of the statistical test). We can calculate Cohen's *d_s_* using:

(17) $$d_{s}=\frac{8.7-7.7}{\sqrt{\frac{\left(10-1\right)0.82^{2}+\left(10-1\right)0.95^{2}}{10+10-2}}}=1.13$$

**Table 3:** **Artificial movie evaluations**.

	**Movie 1**	**Movie 2**	**Difference**
	9.00	9.00	0.00
	7.00	6.00	1.00
	8.00	7.00	1.00
	9.00	8.00	1.00
	8.00	7.00	1.00
	9.00	9.00	0.00
	9.00	8.00	1.00
	10.00	8.00	2.00
	9.00	8.00	1.00
	9.00	7.00	2.00
*M*	8.70	7.70	1.00
*SD*	0.82	0.95	0.67

We can insert this value in G^*^Power to retrieve the estimated sample size needed to find a statistically significant effect in a replication study with α = 0.05, power = 0.95, and an allocation ratio of participants of 1 between conditions. For a two sided test, a power analysis indicates that the estimated sample size would be 44 participants. Finally, remember that a Cohen's *d_s_* of 1.13 is a point estimate. The 95% confidence interval around this effect size estimate can be calculated using a bootstrapping procedure in ESCI (Cumming and Finch, 2005)^1^, which gives 95% CI [0.16, 2.06]. This indicates that although it might be unlikely that people like both movies equally well, we hardly have any idea of how large the difference is. This level of uncertainty should be taken into account when planning the sample size for a study (for alternative approaches to power analysis, see Maxwell et al., 2008).

To report the effect size for a future meta-analysis, we should calculate Hedges's *g* = 1.08, which differs slightly from Cohen's *d_s_* due to the small sample size. To report this study, researchers could state in the procedure section that: “Twenty participants evaluated either Movie 1 (*n* = 10) or Movie 2 (*n* = 10). Participants reported higher evaluations of Movie 1 (*M* = 8.7, *SD* = 0.82) than Movie 2 (*M* = 7.7, *SD* = 0.95), *t*_(18)_ = 2.52, *p* = 0.022, 95% CI [0.17, 1.83], Hedges's *g_s_* = 1.08.” Note that we provide all the necessary statistical information (means, standard deviations, and number of participants in each between-subjects condition). The 95% confidence interval of the difference between the means is provided by default by statistical software packages such as SPSS, but also calculated in the supplementary spreadsheet. Alternatively, you could communicate the uncertainty in the data by providing the 95% confidence interval around the effect size estimate which can be calculated with ESCI (Cumming, 2012). To interpret this effect, we can calculate the common language effect size, for example by using the supplementary spreadsheet, which indicates the effect size is 0.79. We can therefore add the following interpretation of the effect size: “The chance that for a randomly selected pair of individuals the evaluation of Movie 1 is higher than the evaluation of Movie 2 is 79%.”

Now, let's consider a situation where the movie evaluations in Table 3 are collected from the same group of individuals, and each participant has evaluated both movies. Both observations are strongly correlated, with *r* = 0.726. As a consequence, the standard deviation of the difference scores is much smaller than the standard deviations of the evaluations of either movie independently. A dependent *t*-test would provide *t*_(9)_ = 4.74, *p* = 0.001. We can calculate Cohen's *d_z_* using formula 6, but here we calculate the denominator (*S*_diff_) using formula 8:

(18) $$\begin{array}{l} \text{Cohen}\prime \text{s }d_{z}=\frac{1-0}{\sqrt{0.82^{2}+0.95^{2}-2\times 0.726\times 0.82\times 0.95}}\\ \;=1.50 \end{array}$$

This is a markedly higher effect size than Cohen's *d_s_* from the independent *t*-test. Some research questions can only be examined within subjects (see the general discussion), but in this example you might want to be able to compare movie ratings across movies, irrespective of whether all the people who evaluate the movies saw all different movies. Therefore, Hedges's *g_rm_* or Hedges's *g_av_* would provide a more relevant effect size to describe the effect you are interested in. Hedges's *g_av_* is generally recommended (and as the supplementary spreadsheet indicates, also in this specific case), which is 1.08 (note that Hedges's *g_av_* rounds to the same value as Hedges's *g_s_* in the independent *t*-test above).

We can insert Cohen's *d_z_* in G^*^Power to perform an a-priori power analysis to find a statistically significant effect with α = 0.05 and a power of 0.95. For a two sided test the power analysis would indicate a sample size estimate of 8 participants. This clearly demonstrates the dramatic increase in power that a repeated measures design provides if the observations are strongly correlated. This is also reflected in a smaller 95% confidence interval for Cohen's *d_z_* [0.42, 1.80] (for calculations, see ESCI, Cumming and Finch, 2005). To report this study, researchers could write “Ten participants evaluated both Movie 1 and Movie 2. Participants reported higher evaluations of Movie 1 (*M* = 8.7, *SD* = 0.82) than Movie 2 (*M* = 7.7, *SD* = 0.95), *t*_(9)_ = 4.74, *p* = 0.001, 95% CI [0.52, 1.48], Hedges's *g_av_* = 1.08.” The 95% confidence interval of the difference is again by default provided by statistical software packages such as SPSS, as well as provided by the supplementary spreadsheet. Note that we clearly distinguish the way Hedges's *g* is calculated in this study from the way it was calculated in the between-subjects analysis by the subscript. To interpret this result, we can again calculate the common language effect size. For correlated samples, *Z* = *M*_diff_/*S*_diff_ (McGraw and Wong, 1992), and the percentage associated with the upper tail probability of this value is 0.93 (see the supplementary spreadsheet). We can therefore add the interpretation “Controlling for individual differences in movie evaluations, the likelihood that people who watch both movies prefer Movie 1 over Movie 2 is 93%.”

Instead of using *t*-tests, we could have analyzed the data using an analysis of variance (ANOVA). A One-Way ANOVA that mirrors the independent samples *t*-test will provide *F*_(1, 18)_ = 6.34, *p* = 0.022, and statistical software such as SPSS will provide the effect size η^2^_*p*_ = 0.26 (which is identical to η^2^_*G*_ in a between subjects ANOVA). This effect size is identical to the Cohen's *d_s_* of 1.13, as can be seen when we convert Cohen's *d_s_* to *r* using formula 3:

(19) $$r_{pb}=\frac{1.13}{\sqrt{1.13^{2+}\frac{20^{2}-2\times 20}{10\times 10}}}=0.51$$

and since in a One-Way ANOVA *r*^2^ = η^2^_*p*_, 0.51^2^ = 0.26. Inserting η^2^_*p*_ = 0.26 into G^*^Power to perform an a-priori power analysis for two groups, an α = 0.05, and a power of 0.95 will yield a total sample size of 40. This sample size estimate differs from the sample size of 44 that we found for a Cohen's *d_s_* of 1.13. If we would have used Cohen's *d*_pop_ (which is 1.19) the two power analyses would have provided the same sample size estimate of 40. This example highlights a curious state of affairs where researchers (often implicitly) correct for bias in the effect size estimate when they use Cohen's *d_s_*in power analyses, but they do not correct for this bias when they use η^2^_*p*_. To correct for bias ω^2^_*p*_ can be calculated, and although I recommend reporting η^2^_*p*_ or η^2^_*G*_ for practical reasons, calculating ω^2^_*p*_ for simple designs is straightforward. In a One-Way ANOVA with equal sample sizes in each cell, ω^2^_*p*_ can be calculated through the formula:

(20) $$\omega_{p}^{2}=\frac{1\times (5-0.789)}{1\times 5+(20-1)\times 0.789}$$

For the current difference, ω^2^_*p*_ = 0.21, but as explained above, calculating ω^2^_*p*_ can become quite complex in more elaborate designs, and therefore I recommend to report η^2^_*p*_. To report this analysis, researchers could write in the procedure section that: “Twenty participants evaluated either Movie 1 (*n* = 10) or Movie 2 (*n* = 10). Participants reported higher evaluations of Movie 1 (*M* = 8.7, *SD* = 0.82) than Movie 2 (*M* = 7.7, *SD* = 0.95), *F*_(1, 18)_ = 6.34, *p* = 0.022, η^2^_*p*_ = 0.26, 90% CI [0.02, 0.48].” Whereas in a *t*-test, we compare two groups, and can therefore calculate a confidence interval for the mean difference, we can perform *F*-tests for comparisons between more than two groups. To be able to communicate the uncertainty in the data, we should still report a confidence interval, but now we report the confidence interval around the effect size. An excellent explanation of confidence intervals around effect size estimates for *F*-tests, which is accompanied by easy to use syntax files for a range of statistical software packages (including SPSS) is provided by Smithson (2001)^2^. The 90% confidence interval is reported due to the fact that an *F*-test is always a one-sided test, and the 90% confidence interval always excludes 0 when the *F*-test is statistically significant, while the 95% confidence interval does not.

Finally, let's look at the repeated measures ANOVA that mirrors the dependent *t*-test, which gives *F*_(1, 9)_ = 22.50, *p* = 0.001. Statistical software such as SPSS will provide η^2^_*p*_ = 0.71, and using the supplementary spreadsheet we find that η^2^_*G*_ = 0.26 (which is identical to η^2^_*G*_ when analyzing the data as a between-subjects design). For this simple design, we can again easily calculate ω^2^_*p*_:

(21) $$\omega_{p}^{2}=\frac{1\times (5-0.222)}{1\times 5+(10-1)\times 0.222}=0.68$$

We can use η^2^_*p*_ to perform a power analysis. It was already explained that for within-subjects designs, η^2^_*p*_ from SPSS differs from η^2^_*p*_ from G^*^Power. G^*^Power provides two options, “as in SPSS” and “as in Cohen (1988)—recommended.” The difference between the two lies in how the non-centrality parameter (λ) is calculated, which is used in the power calculations. A full explanation of the non-central *t*-distribution is beyond the scope of this article, but for an accessible introduction, see Cumming (2012). The formula either uses *N* (Cohen, 1988) or the degrees of freedom (SPSS). Selecting the “as in SPSS” option will therefore always provide a more conservative estimate. If we select the recommended option “as in Cohen (1988)” G^*^Power returns the estimated sample size of eight participants. Again, readers should be reminded that power analysis provides a point estimate of the minimal sample size, and these calculations should be interpreted while keeping the typical uncertainty about the true effect size in mind.

To report this analysis, researchers could write: “Participants reported higher evaluations for Movie 1 (*M* = 8.7, *SD* = 0.82) than Movie 2 (*M* = 7.7, *SD* = 0.95), *F*_(1, 9)_ = 22.50, *p* = 0.001, η^2^_*p*_ = 0.71, 90% CI [0.31, 0.82], η^2^_*G*_ = 0.26.” Note that I've chosen to report both partial eta squared (including the 90% confidence interval, using the scripts provided by Smithson, 2001) as generalized eta squared. By providing η^2^_*p*_, researchers can perform a-priori power analyses, and by providing η^2^_*G*_, researchers can easily include the study in a future meta-analysis that compares effects across different designs (see Olejnik and Algina, 2003). Providing two effect sizes is in line with the suggestion that reporting multiple effect sizes can yield a greater understanding of a specific effect (Preacher and Kelley, 2011).

## General discussion

The aim of this article was to provide a practical primer on how to calculate and report effect sizes to facilitate cumulative science, with a focus on *t*-tests and ANOVA's. Current practices in the way researchers report effect sizes can be improved. First, researchers should always report effect sizes. When using effect sizes based on Cohen's *d*, researchers should specify which standardizer is used (for example by using subscripts). When reporting effect sizes for ANOVAs it is recommended to report generalized eta squared instead of (or in addition to) partial eta squared. Finally, effect sizes should be interpreted, preferably by comparing them to other effects in the literature or through the common language effect size, instead of using the benchmarks provided by Cohen (1988). This primer explained which effect sizes should be reported and provides a supplementary spreadsheet that researchers can use to easily calculate these effect sizes.

Correctly reporting effect sizes does not only facilitate meta-analyses, but also makes it easier for researchers who build on previous results to perform power analyses. Considering the statistical power of a test when designing a study is useful for cumulative science. As the sample size increases, sampling bias goes down (e.g., Borenstein et al., 2011), and therefore high-powered studies provide better effect size estimates for meta-analyses than studies with low power. Researchers should keep in mind that observed effect sizes in a study can differ from the effect size in the population, and there are reasons to believe overestimations are common given current publication practices where journals mainly accept studies that observe statistically significant effects (Lane and Dunlap, 1978). Early publications of a given finding tend to overestimate the effect size due to regression to the mean (Fiedler et al., 2012). For these reasons, it is inadvisable to focus solely on an a-priori power analysis when the sample size for a future study is determined (unless a very accurate effect size estimate is available), and researchers should pay attention to alternative approaches to plan sample sizes (see Maxwell et al., 2008).

Because power-analyses are inherently tied to null-hypothesis significance testing, some researchers are ambivalent about justifying the sample size of a study based on the likelihood to observe a significant effect. An often heard criticism about null hypothesis significance tests is that the null hypothesis is never true (Schmidt, 1992; Tabachnick and Fidell, 2001). However, the null hypothesis is often a good (and sometimes extremely accurate) approximation (Murphy et al., 2012), and in strictly controlled experiments, it is possible to make the direction of the difference, instead of the size of the effect, central to the purpose of the research (Cohen, 1995). On the other hand, one can reasonably argue that even when researchers are performing a null-hypothesis significance test, they are in reality testing whether an effect is so small that it can be considered negligible (for a detailed description of such minimum-effect tests, see Murphy and Myors, 1999). This in turn requires that researchers at least implicitly consider only effects that are large enough to be theoretically interesting.

The current article is limited to effect sizes for standardized mean differences. Such comparisons are extremely common in experimental psychology, but hardly cover all possible research designs. Instead of a complete overview of effect sizes in experimental research (e.g., Grissom and Kim, 2005), I have tried to provide a practical primer that aims to be an time-efficient but complete overview of one specific type of research question. I therefore see the limitation as a strength, and think similar dedicated overviews for other types of analyses (e.g., risk ratios, multi-level modeling) would be very useful for the scientific community, especially when they are openly accessible. When possible, future articles about effect size calculations should provide software or spreadsheets to make it as easy as possible for researchers to implement these calculations into their workflow. For excellent examples, see ESCI (Cumming and Finch, 2005), confidence interval software by Smithson (2001), and G^*^Power (Faul et al., 2009). Note that the easiest way to facilitate cumulative science is to share the data of the studies you report. The internet makes it incredibly easy to upload data files in order to share them with the scientific community (for example, see www.openscienceframework.org). Especially for mixed designs or analyses with covariates, where calculating ω^2^_*G*_ becomes quite complex, sharing the data will always enable researchers who want to perform a meta-analysis to calculate the effect sizes they need.

A more fundamental question is whether effect sizes from within-subjects designs that control for intra-subjects variability (η^2^_*p*_ and ω^2^_*p*_), or that take the correlation between measurements into account (Cohen's *d_z_*) are an accurate description of the size of the effect, or whether effect sizes that do not control for intra-subjects variability (η^2^_*G*_ and ω^2^_*G*_), or that control for correlation between measurements (e.g., Cohen's *d_rm_* or Cohen's *d_av_*) are preferred. I believe this discussion is currently biased by what could be called *designism*, a neologism to refer to the implicit belief that between-subjects designs are the default experimental design, and that effect sizes calculated from between-subjects designs are more logical or natural. The defense for designism is as follows. It is desirable to be able to compare effect sizes across designs, regardless of whether the observations originate from a within or between-subjects design. Because it is not possible to control for individual differences in between-subject designs, we therefore should consider the effect size that does not control for individual differences as the natural effect size. As a consequence, effect sizes that control for individual differences are “inflated” compared to the “default” (e.g., Dunlap et al., 1996).

Such a reasoning ignores the fact that many effects in psychology are inherently contextual. For example, consider the investigation of how people slow down in a reaction time task after they have made an error (*post-error slowing*; Rabbit, 1966). Recently, Dutilh et al. (2012) have suggested that the best way to answer research questions about post-error slowing is to calculate pairwise comparisons around each error, and analyze these difference scores (against zero, or against the difference score in other conditions), instead of averaging response times over all pre-error and post error responses and compare these two averages in a paired-samples *t*-test. In other words, the difference score is the most natural unit of analysis in such research. Because a between-subjects design is not possible, there will never be a meta-analysis that compares post-error slowing across between and within-subjects designs. Because difference scores are the natural unit of analysis, one could argue that the larger effect sizes are not inflated, but within-subjects analyses simply reflect a different research question, examined at a different level of analysis (intra-individual instead of inter-individual). There are clear parallels with continuing discussions about measures for the proportion of variance explained in multilevel modeling, where it is much more common to assume that repeated measurements of individuals are the default unit of analysis (see Tabachnick and Fidell, 2001).

When empirical questions can only be examined in within-subjects designs (such as in the case of post-error slowing), effect sizes that control for intra-subjects variability (η^2^_*p*_ and ω^2^_*p*_), or that take the correlation between measurements into account (Cohen's *d_z_*) are a reasonable statistic to report. This is nicely demonstrated by the common language effect size (which can be directly calculated from Cohen's *d_s_* or Cohen's *d_z_*). In the illustrative example presented earlier in this article, we concluded the chance that for a randomly selected pair of individuals the evaluation of Movie 1 is higher than the evaluation of Movie 2 is 79% (in the between-subject experiment), but that the chance that an individual who sees both movies (in a within-subject experiment) prefers Movie 1 over Movie 2 is 93%. The CL of 93% is not an overestimation, but an accurate description of the likelihood in correlated samples where measurements are paired. We can calculate effect sizes for within-subject designs (e.g., Cohen's *d_rm_* and Cohen's *d_av_*) that are generalizable to between-subjects designs, but if our goal is to make a statement about whether individuals who watch both movies will prefer Movie 1 over Movie 2, an effect size that generalizes to situations where two different groups of people watch one of the two movies might not provide the best answer to our question.

Generalization across designs (that include or do not include blocking factors, for example) can still be desirable. It would be possible to develop a “within-subjects generalized eta squared” equivalent that excludes variation due to individual differences from the denominator (as η^2^_*p*_) for the effect size calculation, but includes variation due to manipulated factors (as η^2^_*G*_), if one was inclined to make a statement against “designism.” The current article highlights that there is no single “true” definition of an standardized effect size. Researchers need to choose which effect size provides the best summary of the effect, and specify which effect size they report (Thompson, 2007; Cumming, 2012). An efficient way to do so is the use of subscript letters, as used throughout the current article.

In the end, the choice of an effect size calculation depends on the research question and the experimental design. It is important to explicitly state which effect size is calculated, and to make a motivated choice about which effect sizes to report. With the current overview, I hope to have provided a practical primer to assist researchers in choosing and calculating effect sizes, in the conviction that making a more informed choice of about which effect size estimates to report will facilitate cumulative science.

### Conflict of interest statement

The author declares that the research was conducted in the absence of any commercial or financial relationships that could be construed as a potential conflict of interest.

## Footnotes

^1^ESCI can be downloaded from http://www.latrobe.edu.au/psy/research/projects/esci

^2^These files can be downloaded from http://dl.dropboxusercontent.com/u/1857674/CIstuff/CI.html

## Appendix

The parameter Cohen's *f*^2^ used in G^*^Power differs from the parameter for Cohen's *f*^2^ that is used in the statistical software package SPSS. Since η^2^_*p*_ = *f*^2^/1 + *f*^2^, this also means the values for η^2^_*p*_ are not interchangeable between SPSS and G^*^Power. As Erdfelder (personal communication) explains, SPSSη^2^_*p*_ can be converted to G^*^Power η^2^_*p*_ by first converting it to *f*^2^_SPSS_ using:

$$f_{\text{SPSS}}^{2}=\frac{\text{SPSS}\eta_{p}^{2}}{1-\text{SPSS}\eta_{p}^{2}}$$

Then, insert it in the following formula:

$$f_{G*Power}^{2}=f_{\text{SPSS}}^{2}\times \frac{N-k}{N}\times \frac{\left(m-1\right)}{m}\times (1-\rho )$$

where *N* is the sample size, *k* is the number of groups, *m* is the number of repetitions, and ρ is the (mean) correlation between the measures, which can finally be converted into partial eta as it is used in G^*^Power:

$${\text{G}}^{*}\text{Power}\eta_{p}^{2}=f_{{\text{G}}^{*}\text{Power}}^{2}/1+f_{{\text{G}}^{*}\text{Power}}^{2}.$$
